# A Multi-Omics Pan-Cancer Analysis of 4EBP1 in Cancer Prognosis and Cancer-Associated Fibroblasts Infiltration

**DOI:** 10.3389/fgene.2022.845751

**Published:** 2022-03-11

**Authors:** Kunpeng Du, Jingwen Zou, Chunshan Liu, Muhammad Khan, Tao Xie, Xiaoting Huang, Ke Zhang, Yawei Yuan, Baiyao Wang

**Affiliations:** ^1^ Department of Radiation Oncology, Affiliated Cancer Hospital & Institute of Guangzhou Medical University, Guangzhou, China; ^2^ Department of Liver Surgery of the Sun Yat-sen University Cancer Center, Guangzhou, China

**Keywords:** 4EBP1, P-4EBP1, prognosis, cancer-associated fibroblasts, pan-cancer

## Abstract

**Background:** Eukaryotic Translation Initiation Factor 4E Binding Protein 1 (4EBP1) involved in inhibition of protein translation and synthesis. However, the phosphoprotein of 4EBP1 (p-4EBP1) promotes the translation and synthesis of several proteins, including multiple classic oncogenic proteins. The prognostic significance of 4EBP1 mRNA, 4EBP1 protein, and p-4EBP1 in Pan-cancer are still unclear.

**Methods:** In this study, we provided a multi-Omics investigation for the prognostic value of 4EBP1 mRNA, 4EBP1 protein, and different 4EBP1 phosphoproteins in a Pan-cancer manner based on the TCGA projects. We explored the correlation between 4EBP1 expression and the cancer-associated fibroblast (CAFs) infiltration, respectively using the EPIC, MCPCOUNTER, and TIDE algorithms. The functional states of 4EBP1 were explored using single-cell sequencing analysis in Pan-Cancer. Immunohistochemistry staining was used to detect and verify the expression of 4EBP1 in several cancers.

**Results:** 4EBP1 mRNA was aberrantly overexpressed in most cancers, and was associated with the poor prognosis in ten cancers. Notably, increased 4EBP1 mRNA expression significantly correlated with tumor staging and worse prognosis in BRCA, KIRC, and KIRP, while having the opposite effect in STAD. 4EBP1 expression was associated with the CAFs infiltration level in ten cancer types. Interestingly, the correlation between 4EBP1 and CAFs infiltration had pronounced heterogeneity in digestive system tumors and urinary system tumors. In BLCA, KIRC, and ACC as well as BRCA, 4EBP1 was significantly positively correlated with CAFs infiltration and was associated with a poor prognosis. In STAD and COAD, 4EBP1 is negatively correlated with CAFs infiltration and was associated with a better prognosis. Lastly, the expression and prognostic significance of 4EBP1 protein and different p-4EBP1 varied enormously among cancers.

**Conclusion:** Our multi-omics study indicates that 4EBP1-driven CAFs infiltration is associated with cancer prognosis and 4EBP1 mRNA, 4EBP1 protein, and p-4EBP1 proteins may serve as potential prognostic biomarkers and therapeutic targets in diverse cancer.

## Introduction

Eukaryotic Translation Initiation Factor 4E Binding Protein 1 (4EBP1) is a protein-coding gene belonging to the translation repressor proteins family. 4EBP1 is generally considered as a tumor suppressor (Moerke et al., 2007; Martineau et al., 2013; Musa et al., 2016; Wang et al., 2019) involved in the inhibition of the translation and synthesis of multiple classic oncogenic proteins, such as CDK1, HIF-1α, VEGF-A, and MYC (Dodd et al., 2015; Choi et al., 2019; Batool et al., 2020; Haneke et al., 2020). It has been reported that the genetic deletion of 4EBP1 and 4EBP2 significantly accelerate all phases of cancer development in the context of PTEN loss-driven prostate cancer in mice despite potent PI3K/AKT and mTOR activation (Ding et al., 2018). However, when 4EBP1 is phosphorylated by upstream mammalian target of rapamycin (mTOR) signals at specific phosphorylation sites, such as Ser65, Thr37/Thr46, and Thr70, it will liberate the inhibitory effect on protein synthesis (Batool et al., 2020). Therefore, p-4EBP1 was considered to facilitate the tumorigenesis, development, metastasis, and other malignant biological behaviors of multiple tumors, and as an unfavorable prognostic factor in some tumors (Zoncu et al., 2011; Cai et al., 2014).

A recent study indicated that 4EBP1 mRNA and 4EBP1 protein significantly increased in hepatocellular carcinoma tissues., the upregulation of 4EBP1 protein is significantly associated with poor survival and progression (Cha et al., 2015). Nevertheless, another study reported that a high level of p-4EBP1 was involved in prolonging the survival time of patients with gastric cancer (Lee et al., 2015). Therefore, the prognostic significance of 4EBP1 mRNA, 4EBP1 protein, and p-4EBP1 is still controversial (Musa et al., 2016). Importantly, 4EBP1 can be activated by different upstream signals and then phosphorylated at different sites (Qin et al., 2016). A previous study discovered that the levels of 4EBP1 phosphorylated at different sites (T37/46, T70, and S65) varied widely among the different melanoma cell lines and melanoma paraffin specimens (O'Reilly et al., 2009). Moreover, it has been reported that compared to the cap-dependent transcription and protein synthesis function of phosphorylated 4EBP1 at canonical sites (T37, T46, S65, and T70), 4EBP1 phosphorylated at Ser 83 by mitotic protein kinase CDK1 may contribute to cell transformation, which suggested that proteins phosphorylated at different sites may have different functions (Velásquez et al., 2016). Additionally, the higher level of 4EBP1 phosphorylation at Thr70 sites (4EBP1_pT70) in malignant melanoma was associated with a worse prognosis (O'Reilly et al., 2009) and the increase of 4EBP1 phosphorylation at Thr46 sites (4EBP1_pT46) associated with poor prognosis in hepatocellular carcinoma (Lin et al., 2020). Considering that phosphorylation at different sites of 4EBP1 has different effects on the stability, spatial conformation, and function of 4EBP1 protein. It is necessary to explore the expression differences and prognostic significance of different phosphorylated 4EBP1 proteins in cancers.

Given the heterogeneity of tumorigenesis and the complexity of the interconnection and independence of different Omics, we conducted a multi-Omics analysis to evaluate the prognostic value of 4EBP1 mRNA, 4EBP1 protein, and p-4EBP1 proteins in pan-cancer. To further confirm our bioinformatics results, immunohistochemistry staining (IHC) was performed to detect the expression of 4EBP1 in multiple cancers. Moreover, we also explored the potential molecular mechanisms and biological functions of 4EBP1 in the pathogenesis of diverse cancers at the level of immune cell infiltration and single-cell sequencing.

## Methods

### Gene Expression Analysis

We used the Genomic Data Commons Data Portal (GDC; https://portal.gdc.cancer.gov/) to get the latest 10,995 mRNA Seq data of all 33 cancer types from The Cancer Genome Atlas (TCGA) to explore the expression difference of 4EBP1 mRNA level between tumor and normal tissues. As the sequenced data of normal tissues are scarce in the TCGA database, normal human tissues’ expression profile data were obtained from the Genotype-Tissue Expression database (GTEx; https://www.gtexportal.org/) to combine with TCGA data for comparison. mRNA expression values were represented as normalized RNA-Seq by TPM (Transcripts per million) +1. The log2 (TPM +1) was used for log-scale.

Additionally, the mRNA expression of 4EBP1 in different cancer stages (stage I, stage II, stage III, and stage IV) of all TCGA cancer types were downloaded from the UCSC Xena and GSCA portal (http://bioinfo.life.hust.edu.cn/GSCA/#/) (Chen et al., 2019). mRNA sequencing data were processed with RSEM normalized expression and transformed in the log2 (RSEM). The trends of gene expression from stage I to stage IV in different cancers were presented as trend plots.

### Protein Expression Analysis

The UALCAN website (http://ualcan.path.uab.edu/analysis-prot.html) (Chandrashekar et al., 2017; Chen et al., 2019), was used to explore the expression level of the total protein and phosphoproteins (phosphorylation at the Y34, S65, T68, T70, T77, S94, S96, and S101sites) of 4EBP1 between primary tumor and normal tissues, respectively. The protein expression for six available cancer types enrolled in analysis, including colon cancer, breast cancer, ovarian cancer, clear cell renal cell carcinoma, and uterine corpus endometrial carcinoma.

### Gene Survival Analysis

GEPIA2 (http://gepia2.cancer-pku.cn/#index) is a comprehensive web server for systematically analyzing tumor clinical and genomic features across diverse cancer types (Tang et al., 2019). We used the “Survival” module of GEPIA to carry out Cox proportional hazard regression and acquire the Overall survival Kaplan-Meier survival curve of 4EBP1 mRNA across all TCGA tumors. Cutoff-high (50%) and cutoff-low (50%) values were used as the expression thresholds for splitting the high-expression and low-expression groups. The log-rank test was used in the hypothesis test, and a *p*-value ≤0.05 was considered significant.

### Protein Survival Analysis

TRGAted (https://nborcherding.shinyapps.io/TRGAted/) is a web tool enabling researchers to explore the impact of single or multiple proteins on patient survival across 31 cancer types in the TCGA (Borcherding et al., 2018). Therefore, we used it to examine the prognostic value of the total protein and phosphoproteins (phosphorylation at the S65, T70, and T37/T46 sites) of 4EBP1 in Pan-cancer. The median expression value of the protein was selected as the cut-off value to divide the patients into high- and low-expression cohorts in the Kaplan-Meier survival analysis. The optimal cut-off value of the 4EBP1 protein was used in the Cox proportional regression model. The reported *p*-value was based on the log-rank test, while the Hazard Ratio (HR) was calculated for the high-versus-low comparison in the Cox proportional regression model. The log-rank test *p*-value less than 0.05 was considered significant, and the Hazard Ratio of poor prognosis markers was set at 2.0 while the Hazard Ratio of good prognosis markers was set at 0.5.

### Immune Infiltration Analysis

The association between immune cells infiltration, and 4EBP1 expression was estimated by the TIMER2.0 database (http://timer.comp-genomics.org/). TIMER2.0 is a comprehensive web portal providing systematical analysis of immune infiltrates’ abundances estimated by multiple immune deconvolution methods in pan-cancers (Li et al., 2020). In this study, we focused on exploring the correlation between 4EBP1 expression and CAFs infiltration in the tumor microenvironment. Spearman’s correlation based on tumor purity adjustment was used to perform the association analysis. The results were visualized by a heatmap and scatter plots.

### 4EBP1-Related Gene Enrichment Analysis

As a database aimed to annotate functional protein association networks, STRING (https://string-db.org/) collect, integrate, and score almost all publicly available sources of protein-protein interaction (PPI) data (Szklarczyk et al., 2019). We explored the experimentally verified proteins that interacted with 4EBP1 using the STRING website and finally obtained a list of 4EBP1-interacted proteins.

GeneMANIA (http://genemania.org/) is a database to explore the interacted genes with your interested gene through many large, publicly available biological datasets (Warde-Farley et al., 2010). These include co-expression genes, physical interaction genes, and genetic interaction genes. We used the GeneMANIA website to obtain the 20 genes most closely related to 4EBP1.

Then we merged the two gene lists to execute GO (Gene Ontology) enrichment analysis using the R package “clusterProfiler” (Yu et al., 2012). The minimal gene set was set to 15, while the largest gene set was 500. The *p*-value was set to 0.01, and the BH method was used for further multiple test correction for the *p*-value. Then, with q-value ≤ 0.01 as the threshold, the GO term that met this condition was defined as the GO term that significantly enriched the genes’ list.

### Relevance of 4EBP1 Across 14 Functional States in Distinct Cancers

The cancer biology-related functional states of 4EBP1, including angiogenesis, apoptosis, cell cycle, differentiation, DNA damage, DNA repair, EMT, hypoxia, inflammation, invasion, metastasis, proliferation, quiescence, and stemness were estimated at single-cell sequencing level using CancerSEA Portal (http://biocc.hrbmu.edu.cn/CancerSEA/) (Yuan et al., 2019). Correlations between 4EBP1 expression and functional states in different single-cell datasets were screened using correlation strength >0.3 and the P-value < 0.05.

### Immunohistochemistry

Eight tumor types were selected for immunohistochemical staining to experimentally verify the difference in their expression in tumor and normal tissues. These tumor types were selected based on a comprehensive consideration of tumor incidence and the availability of tissue samples. All samples were collected from patients who provided informed consent. The clinicopathological details of the patients were exhibited in Table 1. The patient samples were used with the approval of the internal review and ethics boards of the Affiliated Cancer Hospital and Institute of Guangzhou Medical University. Tumor and normal tissues were fixed with formalin and embedded in paraffin, and finally cut into 5 μm thick sections with a microtome. After dewaxing in xylene and rehydration through graded alcohols to distilled water, the sections were then transferred into sodium citrate solution for boiling to expose the antigen, followed by blocking with normal goat serum. Rabbit anti-4EBP1 antibody (Cell Signaling Technology, Boston, MA, #9644) was diluted at 1:2400 and incubated with the sections at 4°C overnight. Next, a biotinylated goat anti-rabbit IgG secondary antibody was incubated with the sections for 20 min at room temperature. The sections were visualized with the 3, 5-diaminobenzidine (DAB) Substrate Kit which results in a brown-colored precipitate at the antigen site and finally counterstained by Hematoxylin. The staining intensity was scored using a semi-quantitative approach as follows: 0, negative; 1, weak; 2, moderate; and 3, strong. The frequency of positive cells was defined as follows: 0, less than 5%; 1, 5–25%; 2, 26–50%; 3, 51–75%; and 4, greater than 75%. The final IHC scores were obtained by multiplying the staining intensity and the frequency of positive cells. When tissue staining was heterogeneous, each area was scored independently and the scores of each area were added together as the final result.

**TABLE 1:** Baseline clinicopathological characteristics of patients.

Characteristics	HNSC (N = 10)	GBM (N = 10)	LUAD (N = 10)	COAD (N = 10)	LIHC (N = 10)	KIRC (N = 10)	STAD (N = 10)	BRCA (N = 10)
	*n*	*n*	*n*	*n*	*n*	*n*	*n*	*n*
Age								
<50	3	6	4	4	2	3	3	4
≥50	7	4	6	6	8	7	7	6
Sex								
Male	8	7	9	6	8	4	4	0
Female	2	3	1	4	2	6	6	10
Grade								
Grade I/Grade II	4	0	6	7	8	5	4	6
Grade III/Grade IV	6	10	4	3	2	5	6	4
T stage								
T1/T2	2	-	3	2	3	4	2	7
T3/T4	8	-	7	8	7	6	8	3
N stage								
N0	5	-	4	3	5	6	4	9
N1	5	-	6	7	5	4	6	1
M stage								
M0	9	-	10	10	10	10	10	10
M1	1	-	0	0	0	0	0	0

HNSC, head and neck squamous cell carcinoma; GBM, glioblastoma multiforme; LUAD, lung adenocarcinoma; COAD, colon adenocarcinoma; LIHC, liver hepatocellular carcinoma; KIRC, kidney renal clear cell carcinoma; STAD, stomach adenocarcinoma; BRCA, breast invasive carcinoma; -Not Applicable.

### Statistical Analysis

Data are presented as the means ± SD and were analyzed using a student’s t-test or analysis of variance (ANOVA), as appropriate. The correlation expression was analyzed using a Pearson’s chi-squared test. Overall survival curves were plotted using the Kaplan-Meier method and compared using a log-rank test. The Hazard Ratio was calculated by the Cox proportional regression model. All statistical analyses were performed using R 4.02 software, and values of *p* < 0.05 were considered to be statistically significant.

## Results

### Aberrant Expression of 4EBP1 mRNA in Pan-Cancer

We compared the expression level of 4EBP1 mRNA between tumor and adjacent normal tissues in all 33 cancer types in TCGA. The expression level of 4EBP1 in the tumor tissues is higher than the corresponding adjacent normal tissues in almost all tumor types, including BLCA, BRCA, CHOL, COAD, ESCA, GBM, HNSC, KIRC, KIRP, LGG, LIHC, LUAD, LUSC, PRAD, READ, STAD, THCA, and UCEC (*p* < 0.001) (Supplementary Figure S1A). In the Kidney Chromophobe (KICH), the relative expression of 4EBP1 in tumor tissues was significantly reduced. There was no significant difference in the expression of 4EBP1 in pancreatic adenocarcinoma (PAAD) (Supplementary Figure S1A).

By combining the TCGA data with GTEx data, we found the transcriptional levels of 4EBP1 were also significantly elevated in ACC, CESC, LAML, OV, SKCM, TGCT, and UCS compared with corresponding normal tissues. Similarly, we found that the expression of 4EBP1 was elevated in BLCA, BRCA, CHOL, COAD, ESCA, GBM, HNSC, KIRC, KIRP, LGG, LIHC, LUAD, LUSC, PRAD, READ, STAD, THCA, and UCEC and reduced 4EBP1 expression in KICH (Supplementary Figure S1B).

**FIGURE 1:**
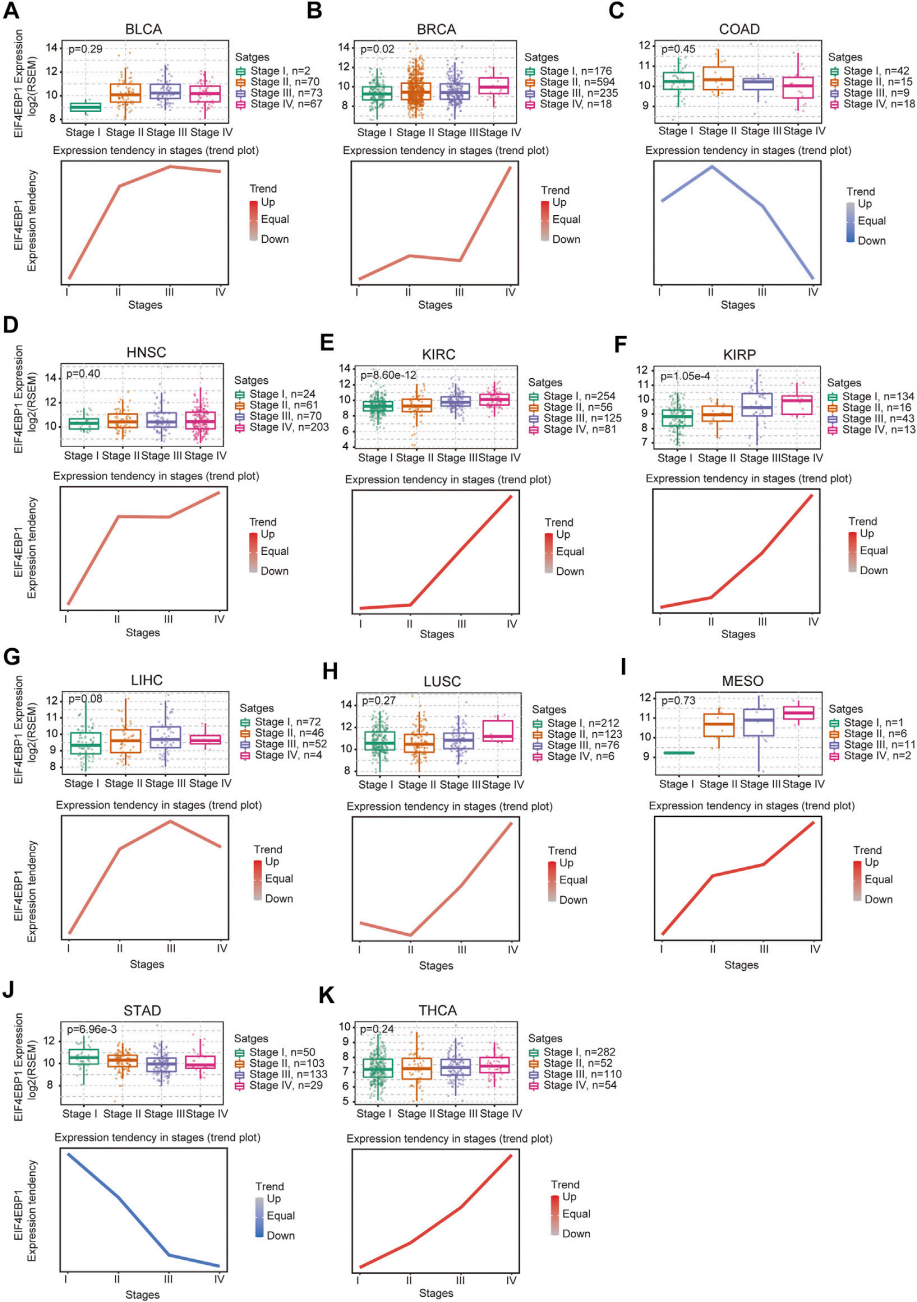
Expression level and trend of 4EBP1 gene in different pathological stages. Based on the TCGA data, the expression levels of the 4EBP1 gene were analyzed by the main pathological stages (Stage I, Stage II, Stage III, and Stage IV) of **(A)** BLCA, **(B)** BRCA, **(C)** COAD, **(D)** HNSC, **(E)** KIRC, **(F)** KIRP, **(G)** LIHC, **(H)** LUSC, **(I)** MESO, **(J)** STAD, **(K)** THCA. Log2 (RSEM) was applied for log-scale.

To explore the potential role of 4EBP1 in tumor progression, we then evaluated the trends of 4EBP1 expression in different pathological stages across all TCGA cancer types and found an increasing tendency in 4EBP1 expression as the tumor progressed in BLCA, BRCA, HNSC, KIRC, KIRP, LIHC, LUSC, MESO, and THCA (Figures 1A,B,D–I,K). These data suggested that 4EBP1 may play a significant role in the progression of these cancers. In contrast, the expression of 4EBP1 exhibits a decreasing tendency as the pathological stage promoting in two digestive system tumors, namely colon adenocarcinoma (COAD) and stomach adenocarcinoma (STAD) (Figures 1C,J).

In summary, 4EBP1 is up-regulated in most cancers, and be associated with the progression of several cancers.

### The Prognostic Value of 4EBP1 mRNA in Pan-Cancer

**FIGURE 2:**
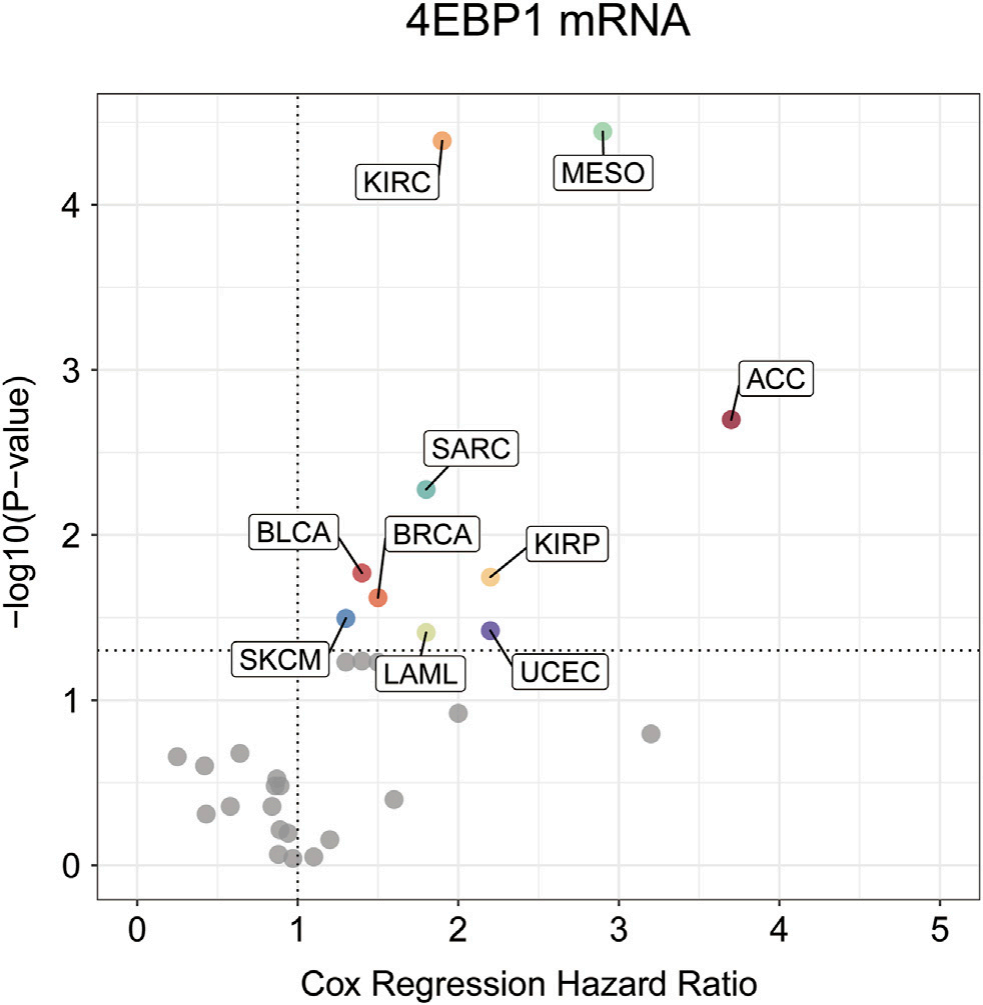
The prognostic significance of 4EBP1 mRNA in Pan-cancer.The survival hazard ratios of 4EBP1mRNA across all 33 cancer types were assessed using the Cox regression model. The cancer types in which 4EBP1 plays as an important prognostic factor (*p*-value<0.05) were displayed with colored dots.

Highly expressed 4EBP1 was associated to worse prognosis of overall survival (OS) among the 10 of 33 cancer types (Figure 2; Supplementary Figure S2), including ACC (*p* = 0.002; HR = 3.7), BLCA (*p* = 0.017; HR = 1.4), BRCA (*p* = 0.024; HR = 1.5), KIRC (*p* = 4.1e-05; HR = 1.9), KIRP (*p* = 0.018; HR = 2.2), LAML (*p* = 0.039; HR = 1.8), MESO (*p* = 3.6e-05; HR = 2.9), SARC (*p* = 0.0053; HR = 1.8), SKCM (*p* = 0.032; HR = 1.3), and UCES (*p* = 0.038; HR = 2.2) within the TCGA project (Supplementary Figures S2B–K). Secondarily, although no statistically significant survival differences were obtained in KICH (*p* = 0.16; HR = 3.2), LIHC (*p* = 0.058; HR = 1.4), and LUAD (*p* = 0.059; HR = 1.3), the higher expression of 4EBP1 still suggested a higher risk of death (Supplementary Figures S2L–N). It is worth noting that in LUSC (HR = 0.87), STAD (HR = 0.86), and UCS (HR = 0.64), the high expression of 4EBP1 mRNA levels predicted a slightly lower risk of death (Supplementary Figures S2O–Q).

### Aberrant Expression of 4EBP1 Total Protein and Phosphorylated Protein in Multiple Cancers

**FIGURE 3:**
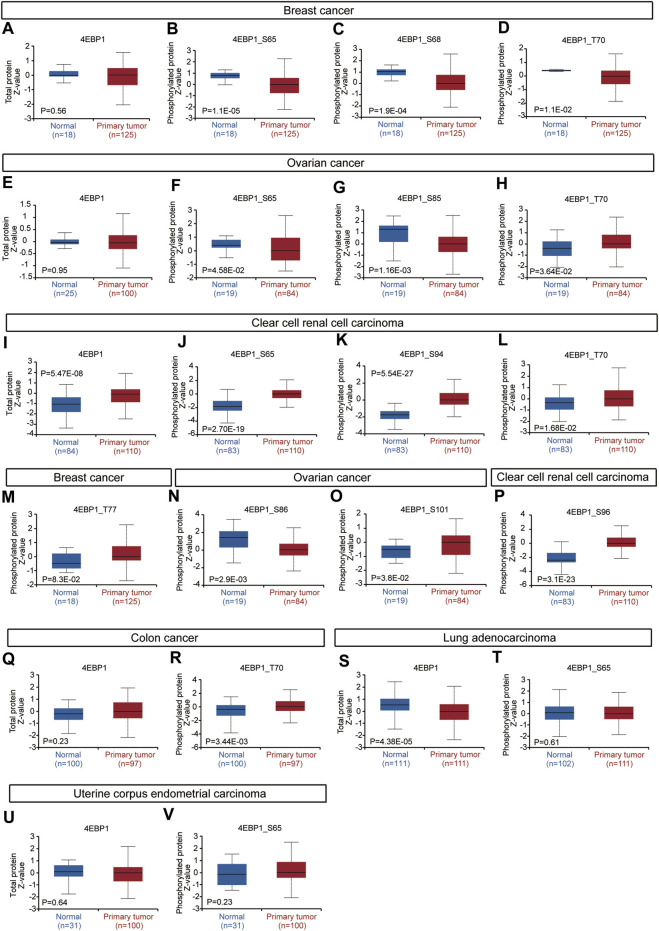
Expression level of 4EBP1 protein and p-4EBP1 phosphoproteins in diverse tumors. Based on the CPTAC dataset, we analyzed the expression level of 4EBP1 total protein and phosphoproteins (S65, S68, T70, T77, S85, S86, S94, S96, and S101 sites) between normal tissue and primary tissue of selected tumors. **(A–D,M)** Breast cancer. **(E–H,N,O)** Ovarian cancer. **(I–L,P)** Clear cell renal cell carcinoma. **(Q,R)** Colon cancer. **(S,T)** Lung adenocarcinoma. **(U,V)** Uterine corpus endometrial carcinoma.

Considering that the 4EBP1 protein and its phosphorylated proteins are executors of biological functions, we further explored the differences in 4EBP1 protein and p-4EBP1 levels between tumor and normal tissues. Compared with normal tissues, the 4EBP1 total protein was significantly up-regulated in clear cell renal cell carcinoma (KIRC) (Figure 3I) and down-regulated in lung adenocarcinoma (LUAD) (Figure 3S). However, no significant expression difference was found in other types of tumors, including breast cancer (Figure 3A), ovarian cancer (Figure 3E), colon cancer (Figure 3Q), and uterine corpus endometrial carcinoma (UCEC) (Figure 3U), which indicated that 4EBP1 involved in biological functions under both normal and tumor conditions. As summarized in Table 2, common phosphorylation sites of 4EBP1, such as S65, S83, T37, T46, and T70, were activated by partially overlapping but not identical upstream kinases. We then evaluated the difference in phosphorylation levels of 4BP1 at different phosphorylation sites between cancer and normal tissues. Compared to normal tissues, 4EBP1_pS65 significantly reduced in breast cancer and ovarian cancer (Figures 3B,F) while elevated in clear cell renal cell carcinoma (Figure 3J). However, there were no significant expression difference of 4EBP1_pS65 in LUAD (Figure 3T) and UCEC (Figure 3V). 4EBP1_pT70 markedly elevated in the tumor tissues of ovarian cancer, clear cell renal cell carcinoma, and colon cancer (Figures 3H,L,R) but decreased in breast cancer (Figure 3D). In addition, 4EBP1_pT68 was observably lower expressed (Figure 3C), and 4EBP1_pT77 was higher expressed in breast cancer tissues (Figure 3M). In ovarian cancer tissues, the levels of 4EBP1_pS85 and 4EBP1_pS86 were lower than in normal tissues (Figures 3G,N), while 4EBP1_pS101 exhibited the opposite result (Figure 3O). In clear cell renal cell carcinoma, the expression of 4EBP1_pS94 and 4EBP1_pS96 dramatically increased compared to normal tissue (Figures 3K,P). The considerable difference in the expression level of different p-4EBP1 made us wonder whether phosphorylated 4EBP1 proteins have different prognostic significance in pan-cancer.

**TABLE 2:** Key upstream kinases at different phosphorylation sites of 4EBP1.

Residue	Site	Upstream Enzymes
T	37	AKT1; ERK2; MAPK14 mTOR
T	46	AKT1; ERK2; MAPK14 mTOR
S	65	AKT1; Ribosomal protein S6 kinase alpha 5; Ras homolog enriched in brain like 1; ERK2; MAPK14; mTOR
T	70	ERK2 mTOR

### The Prognostic Value of the Total Protein and Different Phosphorylated Proteins of 4EBP1 in Pan-Cancer

**FIGURE 4:**
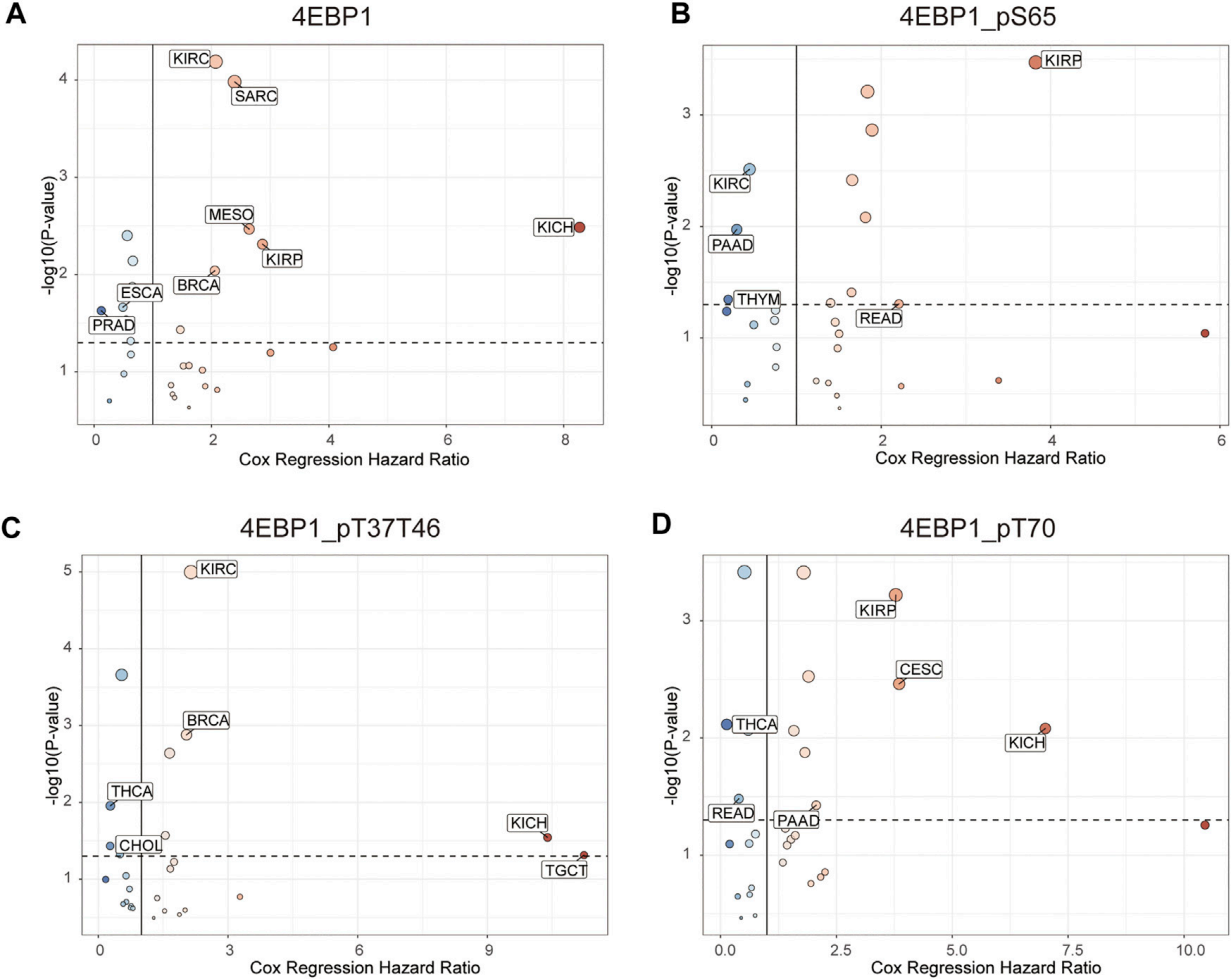
The prognostic significance of 4EBP1 protein, 4EBP1_pS65, 4EBP1_pT37T46, and 4EBP1_pT70 phosphoproteins in Pan-cancer. The survival hazard ratios of **(A)** 4EBP1 protein, **(B)** 4EBP1_pS65, **(C)** 4EBP1_pT37T46, **(D)** 4EBP1_pT70 across all 33 cancer types were assessed using the Cox regression model. The cancer types in which the target protein plays as a poor prognostic factor (HR > 2, *p* < 0.05) were exhibited with pink dots. The cancer types in which the target protein plays as a favorable prognostic factor (HR < 0.5, *p* < 0.05) were displayed with blue dots.

Figure 4 outlines the risk of death (Hazard Ratio, HR) of 4EBP1 protein in Pan-cancer (Figure 4A). The cut-off values of the 4EBP1, 4EBP1_pS65, 4EBP1_pT37/T46, and 4EBP1_pT70 in the Cox proportional regression model and presented in Supplementary Table S1. In KIRC (*p* < 0.0001; HR = 2.07), SARC (*p* < 0.0001; HR = 2.39), MESO (*p* = 0.0026; HR = 2.64), KIRP (*p* = 0.0032; HR = 2.87), KICH (*p* = 0.00048; HR = 8.27), and BRCA (*p* = 0.0077; HR = 2.06), the high expression of 4EBP1 indicated a higher risk of death and an adverse prognosis (Supplementary Figures S3A–F). While in ESCA (*p* = 0.019; HR = 0.49) and PRAD (*p* = 0.0078; HR = 0.123), the high expression of 4EBP1 indicated a lower risk of death and good prognosis (Supplementary Figures S3G,H).

Intriguingly, different 4EBP1 phosphoproteins exhibited distinct effects on the prognosis of different tumors (Figure 4). Highly expressed 4EBP1_pS65 was associated with worse prognosis in KIRP (*p* = 0.00012; HR = 3.83) and READ (*p* = 0.044; HR = 2.21) (Figure 4B; Supplementary Figures S3I,J), while associated with better prognosis in KIRC, PAAD, and THYM (Figure 4B; Supplementary Figures S3K–M). The higher activation level of 4EBP1_pT37T46 was linked to unfavorable prognosis of KIRC (*p* < 0.0001; HR = 2.15), BRCA (*p* = 0.0011; HR = 2.04), KICH (*p* = 0.0065; HR = 10.4) and TGCT (*p* = 0.013; HR = 11.2) (Figure 4B; Supplementary Figures S4A–D). However, the higher activation level of 4EBP1_pT37T46 indicated a lower risk of death and was a favorable prognostic factor in THCA (*p* = 0.0067; HR = 0.28) and CHOL (*p* = 0.026; HR = 0.276) (Figure 4B; Supplementary Figures S4E,F). High level of 4EBP1_pT70 was related to poor prognosis of several cancers, including KIRP (*p* = 0.00023; HR = 3.78), CESC (*p* = 0.0017; HR = 3.85), KICH (*p* = 0.0023; HR = 7.01), and PAAD (*p* = 0.034; HR = 2.06) (Figure 4B; Supplementary Figures S4G–J). However, in THCA (*p* = 0.0017; HR = 0.133) and READ (*p* = 0.028; HR = 0.394), patients with higher level of 4EBP1_pT70 had a higher survival rate (Figure 4B; Supplementary Figures S4K,L). This observation strongly demonstrated that the p-4EBP1 phosphoproteins at S65, T37, T46, and T70 sites had diverse prognostic effects in different tumors, and can be chose as potential prognostic biomarkers in for clinical individualized predictions.

### Correlation Between 4EBP1 and CAFs in the Tumor Microenvironment

**FIGURE 5:**
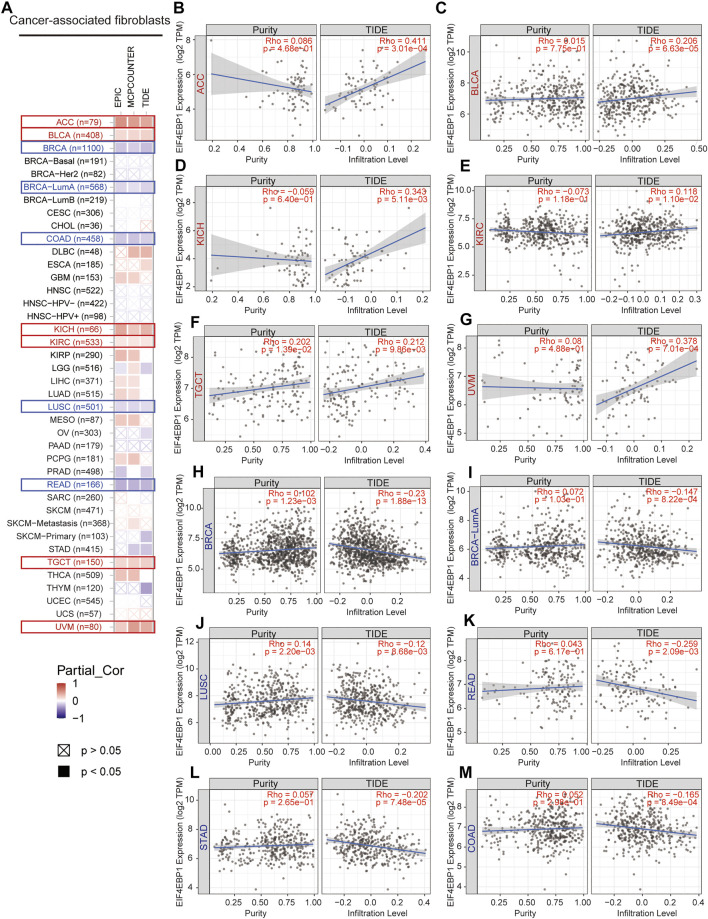
Correlation analysis between 4EBP1 expression and immune infiltration of cancer-associated fibroblasts. **(A)** An overview of the correlation using different algorithms between 4EBP1 expression and CAFs infiltration in Pan-cancer. The positive correlation between the abundance of CAFs and the expression of 4EBP1 in **(B)** ACC, **(C)** BLCA, **(D)** KICH, **(E)** KIRC, **(F)** TGCT, and **(G)** UVM. The negative correlation between the abundance of CAFs and the expression of 4EBP1 in **(H)** BRCA, **(I)** BRCA-LumA, **(J)** LUSC, **(K)** READ, **(L)** STAD, and **(M)** COAD.

CAFs as an essential member of the tumor microenvironment were widely participated in tumor cell growth, metabolic regulation, immune escape, invasion, and metastasis processes (Kalluri, 2016; Sahai et al., 2020). Recent research reported that the mTORC1/4EBP1 axis represents a critical signaling node during fibrogenesis (Woodcock et al., 2019). Herein, we used various algorithms such as EPIC, MCPCOUNTER, and TIDE to investigate the potential correlation between the infiltration level of CAFs and 4EBP1 expression level in multiple cancer types (Figure 5A). Only tumors with coincident correlations of the three algorithms are considered to be significantly related to CAFs infiltration. Finally, we observed that the expression of 4EBP1 was significantly positively correlated with CAFs infiltration in ACC, BLCA, KICH, KIRC, TGCT, and UVM (Figures 5B–G). However, the expression of 4BP1 was significantly negatively correlated with the infiltration of CAFs in BRCA, COAD, LUSC, READ (Figures 5H–L). It is worth noting that in COAD, two algorithms obtained a significant negative correlation between 4EBP1 and CAFs (Figure 5M). The correlation estimated by the TIDE algorithm was exhibited as examples in Figure 5. For example, the expression level of 4EBP1 was positively correlated with the level of infiltration of CAFs in ACC (cor = 0.411, *p* = 3.01e-04).

### Enrichment Analysis of 4EBP1-Related Partners

**FIGURE 6:**
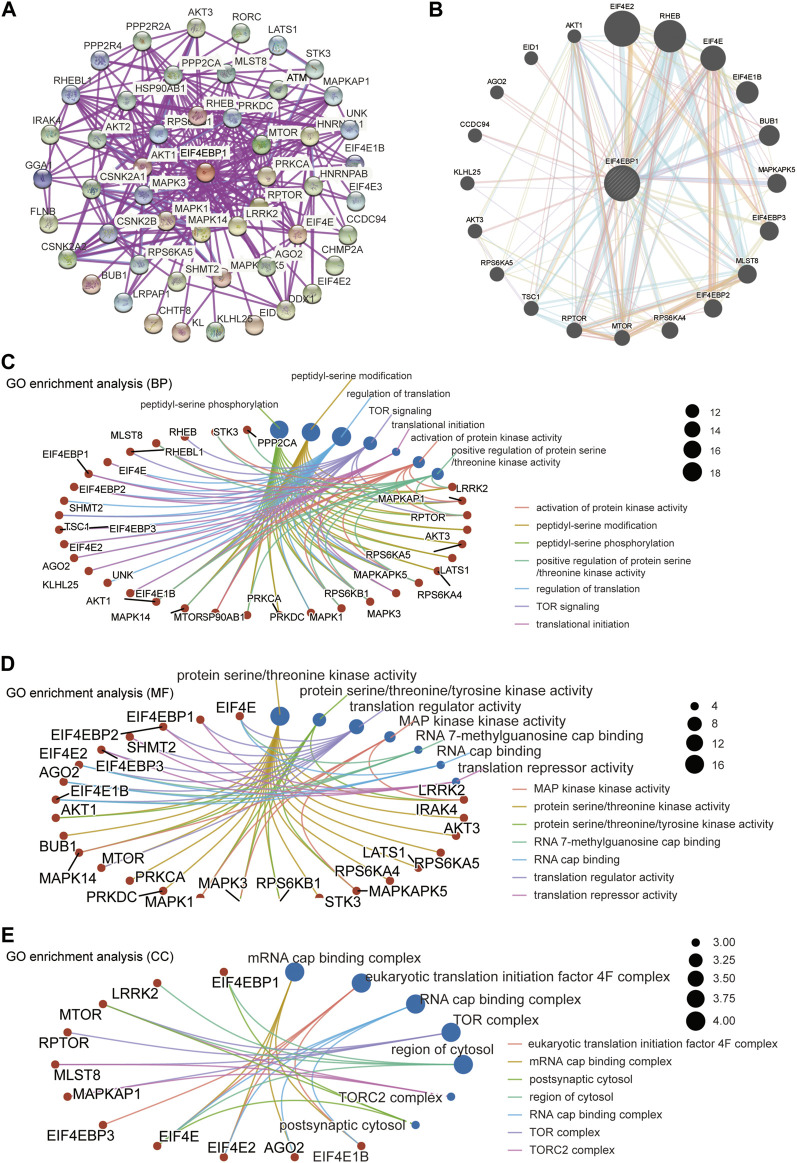
4EBP1-related gene enrichment analysis. **(A)** We obtained the available experimentally determined 4EBP1-binding proteins using the STRING database. **(B)** We used the GeneMANIA website to get the 20 genes most closely related to 4EBP1. **(C)** The biological process, **(D)** molecular function, and **(E)** cell components involved in 4EBP1 in GO enrichment analyses.

A total of 50 proteins that interact with 4EBP1, verified by experimental evidence, were obtained through the STRING database. The protein interaction network was shown in Figure 6A. On the GeneMANIA website, twenty proteins that interact with 4EBP1 were discovered and displayed in Figure 6B.

We combined two sets of gene lists to perform GO enrichment analysis to reveal the biological process (BP), molecular function (MF), and cellular component (CC) involved in 4EBP1. The biological process involved in 4EBP1 and its related genes covered the regulation of translation, TOR signaling, and translational initiation (Figure 6C). The main biological functions of 4EBP1 included translation regulator activity and translation repressor activity (Figure 6D). The cell components related to 4EBP1 include postsynaptic cytosol and region of cytosol l (Figure 6E).

### Functional States of 4EBP1 Across Different Cancer Types

The functional states of 4EBP1 were analyzed in 16 cancers, including LUAD, non-small cell lung cancer (NSCLC), renal cell carcinoma (RCC), BRCA, head, and neck cancer (HNSCC), OV, and colorectal cancer (CRC) (Supplementary Figure S5). 4EBP1 was negatively correlated with differentiation (cor = -0.391, *p* = 0.011) and quiescence (cor = -0.433, *p* = 0.003) in LUAD. 4EBP was positively associated with the cell cycle (cor = 0.306, *p* < 0.001) and DNA damage (cor = 0.33, p = < 0.001), while negatively with metastasis (cor = -0.317, p = < 0.001) in NSCLC. In BRCA, 4EBP1 was positively correlated with the cell cycle (cor = 0.363, *p* < 0.001), DNA repair (cor = 0.45, *p* < 0.001), and invasion (cor = 0.538, *p* = 0.005). 4EBP1 expression was positively correlated with angiogenesis (cor = 0.36, *p* = 0.008) and hypoxia (cor = 0.289, *p* = 0.009), while negatively with DNA repair (cor = -0.30, *p* = 0.007) and invasion (cor = -0.522, *p* = 0.008) in OV. In CRC, the expression of 4EBP1 was positively correlated with angiogenesis (cor = 0.36, *p* = 0.008) and inflammation (cor = 0.34, *p* = 0.011) (Supplementary Figure S5).

### Expression of 4EBP1 in Clinical Specimens of Multiple Cancers

**FIGURE 7:**
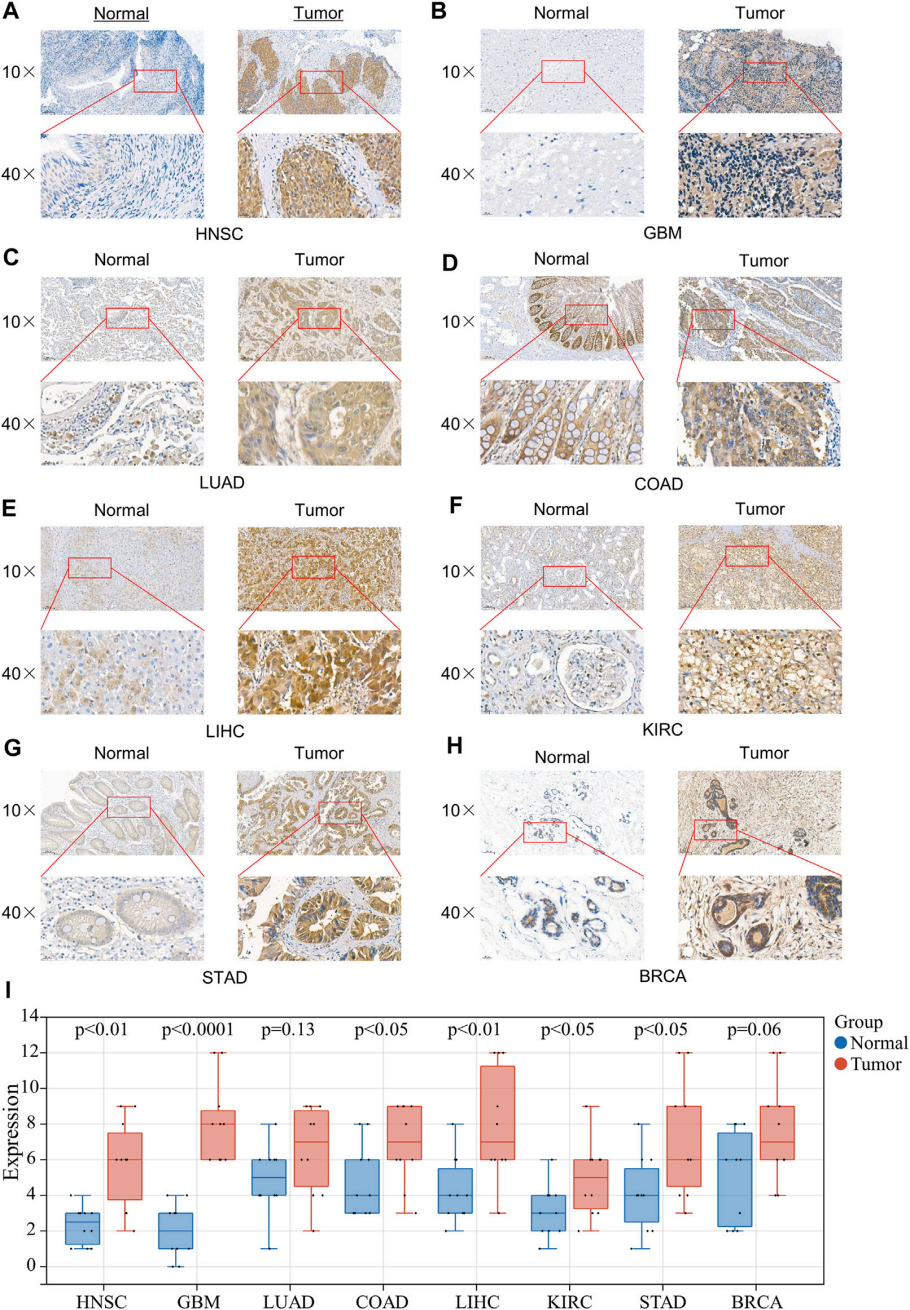
Expression of 4EBP1 in clinical specimens of multiple cancers. Representative images of 4EBP1 (brown, cell cytoplasmic/nucleus stain) IHC of **(A)** HNSC (Nasopharyngeal Carcinoma), **(B)** GBM, **(C)** LUAD, **(D)** COAD, **(E)** LIHC, **(F)** KIRC, **(G)** STAD, and **(H)** BRCA. **(I)** IHC quantification and comparison results between cancer and adjacent tissues (*t*-test).

To further verify the expression results obtained by our bioinformatics, we assessed the expression of 4EBP1 in tumor and adjacent tissues of a variety of tumors using IHC, including HNSC, GBM, LUAD, COAD, LIHC, KIRC, STAD, and BRCA (Figure 7). As expected, 4EBP1 was significantly elevated in HNSC and GBM, COAD, LIHC, KIRC, and STAD compared to adjacent tissues (*p* < 0.05) (Figure 7I). In LUAD and BRCA, 4EBP1 was increased in tumor tissues but not statistically significant (Figure 7I).

## Discussion

To our knowledge, there was no literature on the potential prognostic impact and biological function of 4EBP1 in Pan-cancer. Moreover, no research focused on the prognostic value of 4EBP1 at the level of transcriptomics, proteomics, and phosphorylation proteomics in Pan-cancer. In this study, we found that 4EBP1 mRNA was up-regulated in almost all cancers. These results go along with a previous study that 4EBP1 mRNA and protein are markedly up-regulated in Hepatocellular carcinoma tissues (Cha et al., 2015). Moreover, we found that the expression of 4EBP1 exhibited a progressive trend with the increase of tumor stage in BLCA, BRCA, HNSC, KIRC, KIRP, LIHC, LUSC, MESO, and THCA. These conspicuous results indicated that 4EBP1 might contribute to the progression of these cancers. Coincidentally, in COAD and STAD, which are both gastrointestinal tumors, the expression of 4EBP1 decreased with the increase of tumor stage. These interesting results seem to imply that 4EBP1 may play a suppressive role in tumor progression in certain gastrointestinal tumors.

4EBP1 expression was correlated with poor prognosis in 10 tumor types, including ACC, BLCA, BRCA, KIRC, KIRP, LAML MESO, SARC, SKCM, and UCES. These results further confirmed that 4EBP1 might act as an adverse prognostic biomarker for BLCA, BRCA, KIRC, KIRP, and MESO because the increased expression of 4EBP1 was related to both the staging and prognosis of these tumors.

It was worth noting that in LUSC, STAD, and UCS, patients with high expression of 4EBP1 were slightly associated with better overall survival. This result partially answered the above question, that 4EBP1 may act as a tumor suppressor in STAD and may serve as a favorable prognostic marker for STAD.

Considering that proteins are executors of biological functions, we further explored the expression differences and the prognostic values of 4EBP1 and p-4EBP1 proteins in a variety of cancers. Compared with adjacent normal tissues, the expression level of 4EBP1 protein was significantly up-regulated in KIRC and down-regulated in LUAD, but there is no significant difference in other types of tumors. Phosphoprotein 4EBP1_pS65 was elevated considerably in KIRC while reduced in BRCA and OV. 4EBP1_pT70 was markedly elevated in the tumor tissues of KIRC, OV, and COAD but decreased in BRCA. The enormous difference in the expression level of 4EBP1 phosphoproteins was strongly speculated related to different prognostic significance in diverse cancer types. To further verify the elevated expression of 4EBP1 in tumor tissues, we also performed immunohistochemical staining on pathological sections of a variety of tumors. In the eight tumor types we performed immunohistochemistry, all 4EBP1 protein levels were increased, among which HNSC, GBM, COAD, LIHC, KIRC, and STAD were significantly increased, while LUAD and BRCA were slightly increased. These results were in line with the 4EBP1 expression results we obtained from TCGA.

Survival analysis results indicated that the high expression of 4EBP1 protein was associated with a higher risk of death and worse prognosis of patients with KIRC, SARC, MESO, KIRP, KICH, BRCA, and SARC. Then, we performed a pan-cancer analysis of the prognosis of different 4EBP1 phosphoproteins using large-scale proteomics sequencing data and clinical data for the first time. Interestingly, different 4EBP1 phosphoproteins had different effects on the prognosis of different cancer types. Highly expressed 4EBP1_pS65 was significantly correlated with worse overall survival in KIRP, and READ, while was correlated with better survival in KIRC, PAAD, and THYM. The higher level of 4EBP1_pT37T46 was associated with poor prognosis in KICH, KIRC, and BRCA but was a favorable prognosis in THCA and CHOL. In addition, higher expressed 4EBP1_pT70 was significantly correlated with worse overall survival in KIRP, CESC, KICH, and PAAD. However, in READ and THCA, patients with a higher level of 4EBP1_pT70 had a higher overall survival rate. Our multi-Omics survival analysis suggested that 4EBP1 protein and different p4EBP1 had different prognostic effects. For example, the high level of 4EBP1 expression and 4EBP1_pT37T46 of KIRC patients indicated a worse prognosis, while a high level of 4EBP1_pS65 indicated a better prognosis. In READ, a high level of 4EBP1_pS65 was associated with worse survival, while a high level of T70 is associated with better survival. In KIRP, high expression of 4EBP1, 4EBP1_pS65, and 4EBP1_pT70 were all related to the worse prognosis of patients. Therefore, in clinical practice, we can more accurately assess the prognosis of patients based on the comprehensive analysis of 4EBP1 and different p4EBP1 levels. In previous studies, Qu and others proved that p-4EBP1 was associated with poor prognosis in renal cell carcinoma (Qu et al., 2016). Nishikawa et al. demonstrated that the expression level of p-4EBP1 was significantly correlated with worse survival in patients with metastatic renal cell carcinoma (Nishikawa et al., 2014). O’Reilly et al. found that phosphorylated 4EBP1 was associated with poor survival in melanoma (O'Reilly et al., 2009). However, previous studies focused on the prognostic value of total phosphorylated 4EBP1. Our study made up for the lack of research on the prognostic effect of different p-4EBP1 proteins.

Recently, increasing evidence had demonstrated that CAFs, as a prominent component of the tumor microenvironment (TME), could affect tumor initiation, progression, immune escape, metastasis and act as an essential determinant of immunotherapy response and clinical outcome (Sahai et al., 2020), (Bussard et al., 2016; Steven and Seliger, 2018; Chen and Song, 2019). Our findings firstly discovered the association of 4EBP1 expression and infiltration level of CAFs in diverse tumors. The expression of 4EBP1 was significantly positively correlated with CAFs infiltration in ACC, BLCA, KICH, KIRC, TGCT, and UVM. However, the expression of 4BP1 in BRCA, COAD, LUSC, READ, and STAD was significantly negatively correlated with the infiltration of CAFs. Here, we also found an intriguing phenomenon. In a variety of urinary system tumors, such as bladder urothelial carcinoma (BLCA) and kidney cancer (KICH and KIRC), 4EBP1 was positively correlated with the degree of CAFs infiltration, while in digestive system malignancy, such as colon adenocarcinoma (COAD), rectum adenocarcinoma (READ) and stomach adenocarcinoma (STAD), it was negatively correlated. Given CAFs were recognized to promote tumorigenesis and development in most cases, a higher degree of CAFs immune infiltration was considered to be related to a worse prognosis of patients (Gieniec et al., 2019; Hosein et al., 2020; Piersma et al., 2020). Therefore, combining the above prognostic data, our results explained that in ACC, BLCA, and KIRC, higher 4EBP1 levels led to poor prognosis, which may be related to the higher infiltration of CAFs in TME. Conversely, in LUSC and STAD, higher 4EBP1 levels led to a lower degree of immune infiltration of CAFs, which in turn led to better survival. In summary, our study strongly suggests that 4EBP1 may affect the prognosis of patients by affecting cancer-associated fibroblasts infiltration in a variety of tumors. CAFs seem to bridge 4EBP1 expression and prognosis, which is a new direction for exploring the potential mechanism of tumor immune escape and can guide the development of therapeutic methods for the immune microenvironment.

The results of GO enrichment analysis emphasized the vital role of 4EBP1 in translation regulation and protein synthesis. The functional status of 4EBP1 revealed that it might be related to angiogenesis, DNA repair, apoptosis, cell cycle, and tumor invasion. The above results once again confirmed the behavior of 4EBP1 is a critical molecule in protein synthesis and revealed the malignant biological processes in 4EBP1 participation. These all suggested that 4EBP1, especially p-4EBP1, may serve as potential prognostic biomarkers and therapeutic targets in diverse cancer types.

However, our study has some limitations. The prognostic effects of different p-4EBP1 need to be proved in more extensive clinical cohorts. The correlation between 4EBP1 and CAFs immune infiltration still needs to be verified by *in vitro* or *in vivo* experiments.

## Conclusion

Our multi-Omics studies revealed that 4EBP1 expression was correlated with tumor staging, clinical prognosis, protein phosphorylation, CAFs infiltration, and cancer biology-related functions across multiple tumors, which helps in comprehending the role of 4EBP1 in tumorigenesis and progression from numerous perspectives. More importantly, we found that 4EBP1 protein and p-4EBP1 had different prognostic effects on different cancers, contributing to the development of more precise targeted therapy drugs. Therefore, 4EBP1 mRNA, 4EBP1 protein, and p-4EBP1 proteins may play vital roles in tumor immunity and serve as potential prognostic biomarkers as well as therapeutic targets in diverse cancer.

## Data Availability

The original contributions presented in the study are included in the article/Supplementary Material, further inquiries can be directed to the corresponding authors.

## Supplementary Material

Supplementary Figure S1 4EBP1 is abnormally expressed in human Pan-cancer. **(A)**Differential expression of 4EBP1 in cancers and normal tissues from TCGA database. **(B)** 4EBP1 is aberrant overexpressed in 25 cancer types from the TCGA database and GTEx database (*P < 0.05, **P < 0.01, ***P < 0.001).

Supplementary Figure S2 Correlation between 4EBP1 mRNA expression and survival prognosis of cancers in TCGA. The overall survival curves of 4EBP1 mRNA in different cancers. **(A)** The survival map exhibits the survival significance of 4EBP1 in pan-cancer. Kaplan-Meier curves with positive results are given, including **(B)** ACC, **(C)** BLCA, **(D)** BRCA, **(E)** KIRC, **(F)** KIRP, **(G)** LAML, **(H)** MESO, **(I)** SARC, **(J)** SKCM, and **(K)** UCEC. Kaplan-Meier curves with negative but meaningful results are exhibited, including **(L)** KICH, **(M)** LIHC, **(N)** LUAD, **(O)** LUSC, **(P)** STAD, and **(Q)** UCS.

Supplementary Figure S3 Kaplan-Meier curves of 4EBP1 protein and 4EBP1_pS65 phosphoprotein in cancers. Kaplan-Meier survival curves of 4EBP1 protein in statistically significant tumor types, including **(A)** KIRC, **(B)** SARC, **(C)** MESO, **(D)** KIRP, **(E)** KICH, **(F)** BRCA, **(G)** ESCA, and **(H)** PRAD. Kaplan-Meier survival curves of 4EBP1_pS65 protein in statistically significant tumor types, including **(I)** KIRP, **(J)** READ, **(K)** THYM, **(L)** KIRC, and **(M)** PAAD.

Supplementary Figure S4 Kaplan-Meier curves of 4EBP1_pT37T46 and 4EBP1_pT70 phosphoprotein in cancers. Kaplan-Meier survival curves of 4EBP1_pT37T46 in statistically significant tumor types, including **(A)** KIRC, **(B)** BRCA, **(C)** KICH, **(D)** TGCT, **(E)** THCA, **(F)** CHOL. Kaplan-Meier survival curves of 4EBP1_pT70 in statistically significant tumor types, including **(G)** KIRP, and **(H)** CESC **(I)** KICH, **(J)** PAAD, **(K)** THCA, and **(L)** READ.

Supplementary Figure S5 The functional state of 4EBP1 across 16 types of cancer. The red plots indicated that 4EBP1 is positively correlated with the functional state while the blue plots indicate that 4EBP1 was negatively correlated with the functional state identified by CancerSEA.

## Glossary

4EBP1: eukaryotic translation initiation factor 4E binding protein 1
ACC: adrenocortical carcinoma
BLCA: bladder urothelial carcinoma
BRCA: breast invasive carcinoma
BP: biological process
CancerSEA: cancer single-cell state atlas
CAFs: cancer-associated fibroblasts
CESC: cervical squamous cell carcinoma and endocervical adenocarcinoma
CHOL: cholangiocarcinoma
CC: cellular component
COAD: colon adenocarcinoma
CPTAC: clinical proteomic tumor analysis consortium;
DLBC: lymphoid neoplasm diffuse large B-cell lymphoma
ESCA: esophageal carcinoma
GTEx: genotype tissue expression database
GDC: genomic data commons data portal
GBM: glioblastoma multiforme
GSCA: gene set cancer analysis
HR: hazard ratio
HNSC: head and neck squamous cell carcinoma
KICH: kidney chromophobe
KIRC: kidney renal clear cell carcinoma
KIRP: kidney renal papillary cell carcinoma
LAML: acute myeloid leukemia
LGG: lower grade glioma
LIHC: liver hepatocellular carcinoma
LUAD: lung adenocarcinoma
LUSC: lung squamous cell carcinoma
OV: ovarian serous cystadenocarcinoma
PAAD: pancreatic adenocarcinoma
PRAD: prostate adenocarcinoma
READ: rectum adenocarcinoma
SKCM: skin cutaneous melanoma
STAD: stomach adenocarcinoma
TGCT: testicular germ cell tumors
THCA: thyroid carcinoma
UCEC: uterine corpus endometrial carcinoma
UCS: uterine carcinosarcoma
MESO: mesothelioma
PCPG: pheochromocytoma and paraganglioma
SARC: sarcoma
TPM: transcripts per million
THYM: thymoma
TCGA: the cancer genome atlas
UVM: uveal melanoma
MF: molecular function
