# Supplementary material for: A Multi-Omics Pan-Cancer Analysis of 4EBP1 in Cancer Prognosis and Cancer-Associated Fibroblasts Infiltration
Source: Front Genet. 2022 Mar 11;13:845751. doi: 10.3389/fgene.2022.845751 (PMC8963376; doi:10.3389/fgene.2022.845751)
Supplement: Supplementary file 1 [file Table1.docx]

| **Supplemental Table1. The optimal cut-off value of the 4EBP1, p-4EBP1 proteins in the Cox proportional regression model** | | | | | | | |
| --- | --- | --- | --- | --- | --- | --- | --- |
| **Cancer type** | **Optimal cut-off value** | **Optimal cut-off value**  **（Z-score）** | **Number of low-group** | **Number of high-group** | **HR** | **P-value** | **log10P** |
| **4EBP1** | | | | | | | |
| KIRC | 0.43 | -3.99 | 371.00 | 73.00 | 2.07 | 0.00 | 4.19 |
| SARC | 0.00 | -3.88 | 122.00 | 99.00 | 2.39 | 0.00 | 3.98 |
| MESO | 0.03 | -2.93 | 16.00 | 44.00 | 2.64 | 0.00 | 2.47 |
| KIRP | -0.39 | -2.82 | 124.00 | 82.00 | 2.87 | 0.00 | 2.31 |
| KICH | -0.21 | -2.94 | 51.00 | 11.00 | 8.27 | 0.00 | 2.49 |
| BRCA | 0.32 | -2.61 | 177.00 | 696.00 | 2.06 | 0.01 | 2.04 |
| ESCA | 0.04 | 2.30 | 55.00 | 71.00 | 0.49 | 0.02 | 1.66 |
| PRAD | -0.43 | 2.26 | 52.00 | 299.00 | 0.12 | 0.02 | 1.63 |
| **4EBP1_pS65** | | | | | | | |
| KIRP | -0.05 | -3.58 | 169.00 | 37.00 | 3.83 | 0.00 | 3.47 |
| READ | -0.05 | -1.96 | 99.00 | 27.00 | 2.21 | 0.05 | 1.31 |
| THYM | -0.04 | 2.00 | 29.00 | 60.00 | 0.19 | 0.05 | 1.35 |
| PAAD | -0.51 | 2.55 | 88.00 | 17.00 | 0.30 | 0.01 | 1.97 |
| KIRC | 0.08 | 2.96 | 374.00 | 70.00 | 0.45 | 0.00 | 2.51 |
| **4EBP1_pT37T46** | | | | | | | |
| KIRC | 0.52 | -4.42 | 348.00 | 96.00 | 2.15 | 0.00 | 5.00 |
| BRCA | 0.67 | -3.21 | 742.00 | 131.00 | 2.04 | 0.00 | 2.88 |
| KICH | -0.70 | -2.19 | 35.00 | 27.00 | 10.39 | 0.03 | 1.54 |
| TGCT | 0.73 | -1.97 | 87.00 | 17.00 | 11.24 | 0.05 | 1.31 |
| THCA | -1.12 | 2.54 | 70.00 | 302.00 | 0.28 | 0.01 | 1.96 |
| CHOL | -1.10 | 2.09 | 6.00 | 24.00 | 0.28 | 0.04 | 1.43 |
| **4EBP1_pT70** | | | | | | | |
| KIRP | -0.18 | -3.43 | 128.00 | 78.00 | 3.78 | 0.00 | 3.22 |
| CESC | 0.13 | -2.92 | 66.00 | 105.00 | 3.85 | 0.00 | 2.46 |
| KICH | 0.05 | -2.64 | 52.00 | 10.00 | 7.01 | 0.01 | 2.08 |
| PAAD | -0.17 | -2.08 | 27.00 | 78.00 | 2.06 | 0.04 | 1.43 |
| THCA | -0.27 | 2.67 | 191.00 | 181.00 | 0.13 | 0.01 | 2.12 |
| READ | -0.16 | 2.13 | 69.00 | 57.00 | 0.39 | 0.03 | 1.48 |
