# Supplementary figures and images for: A Multi-Omics Pan-Cancer Analysis of 4EBP1 in Cancer Prognosis and Cancer-Associated Fibroblasts Infiltration
Source: Front Genet. 2022 Mar 11;13:845751. doi: 10.3389/fgene.2022.845751 (PMC8963376; doi:10.3389/fgene.2022.845751)

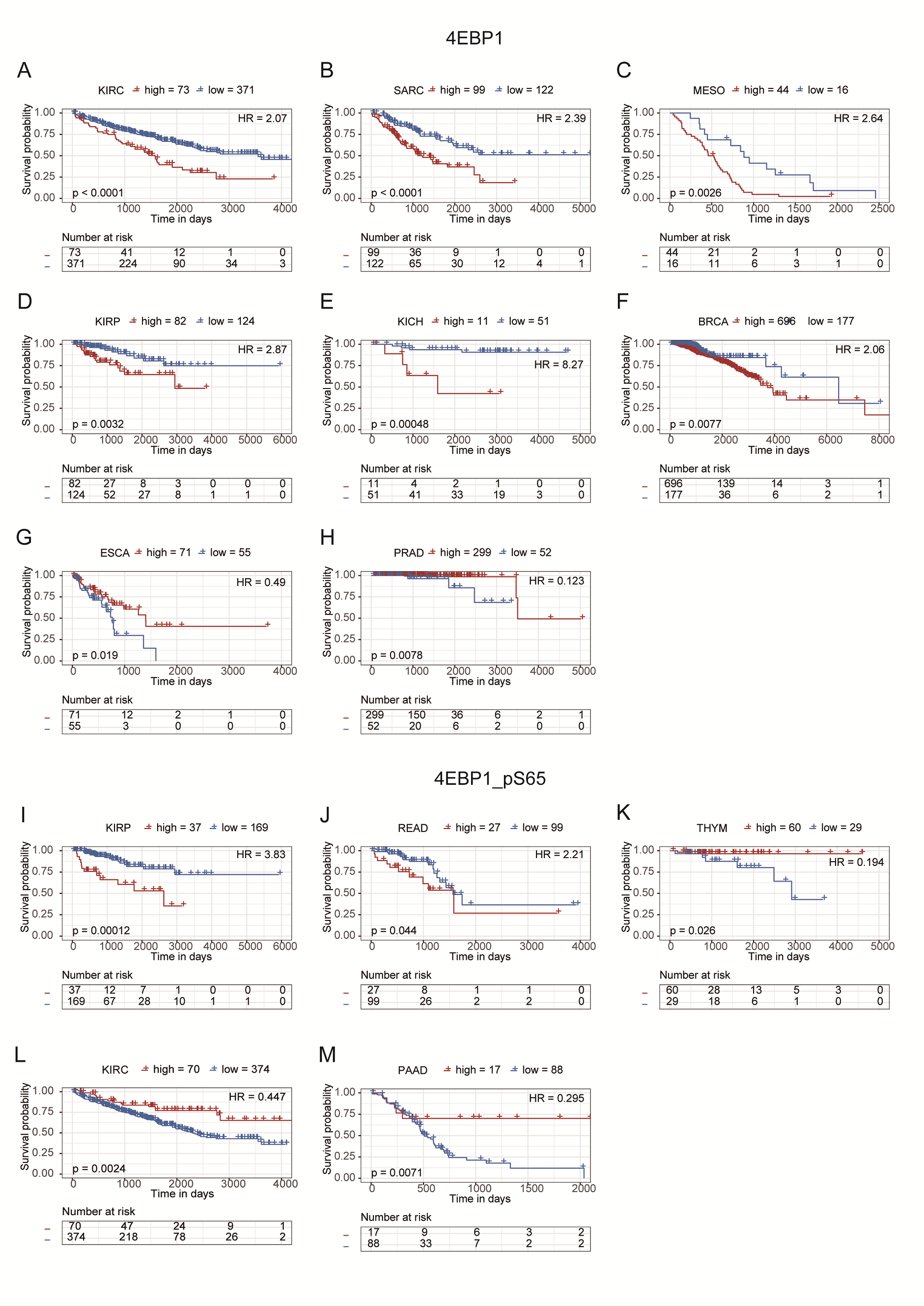

Supplement: Supplementary file 2 [file Image3.TIF]

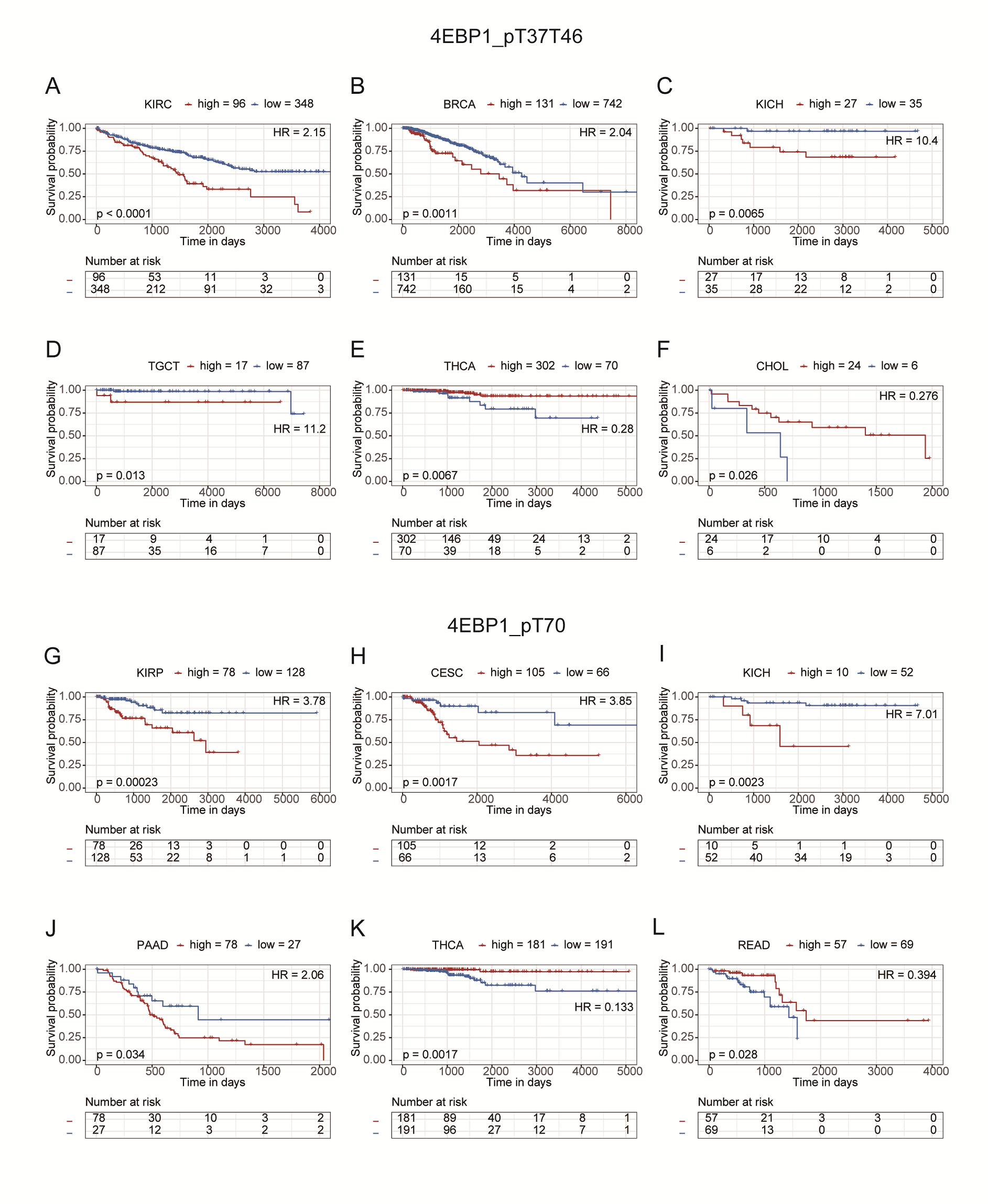

Supplement: Supplementary file 3 [file Image4.TIF]

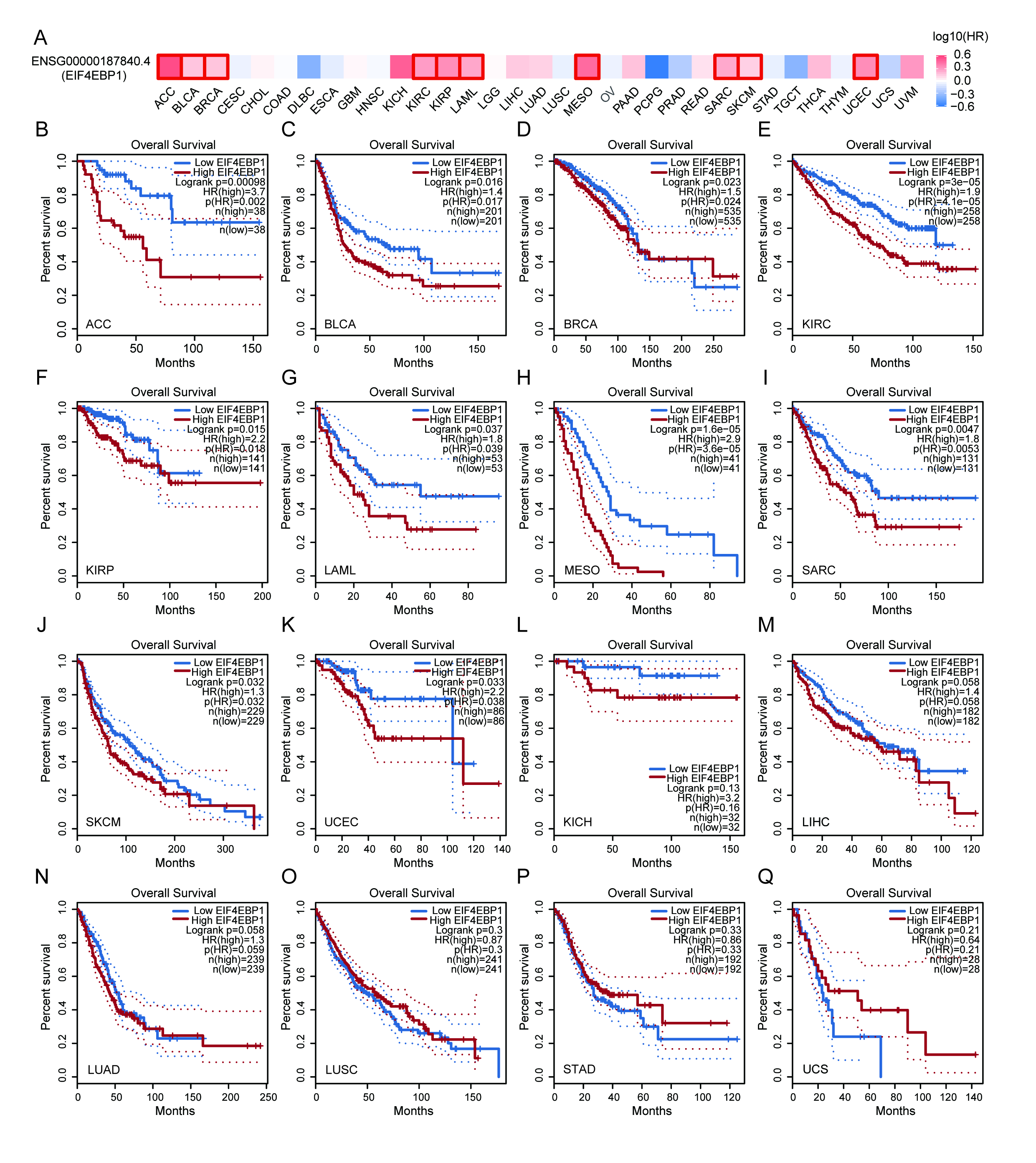

Supplement: Supplementary file 4 [file Image2.TIF]

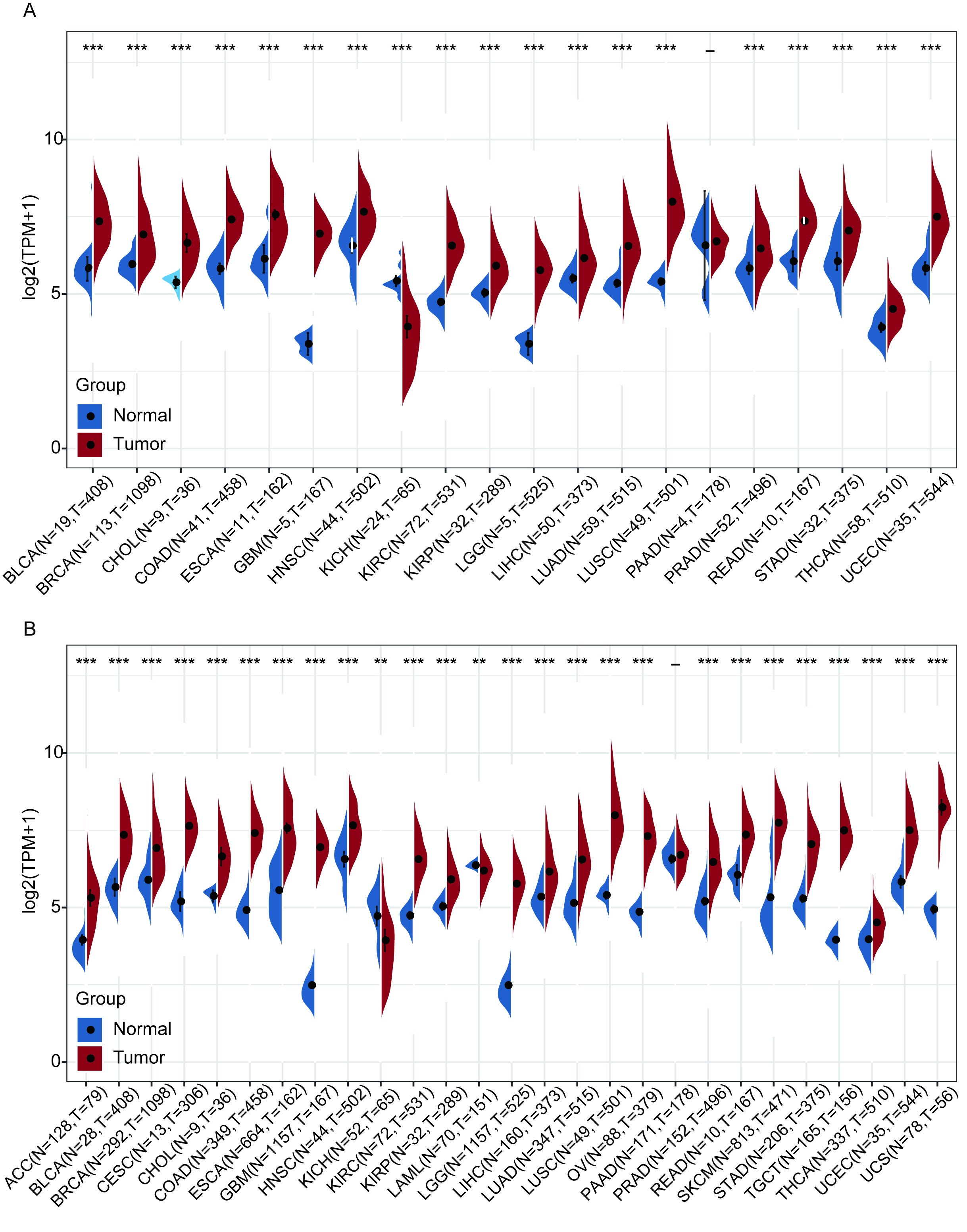

Supplement: Supplementary file 5 [file Image1.TIF]

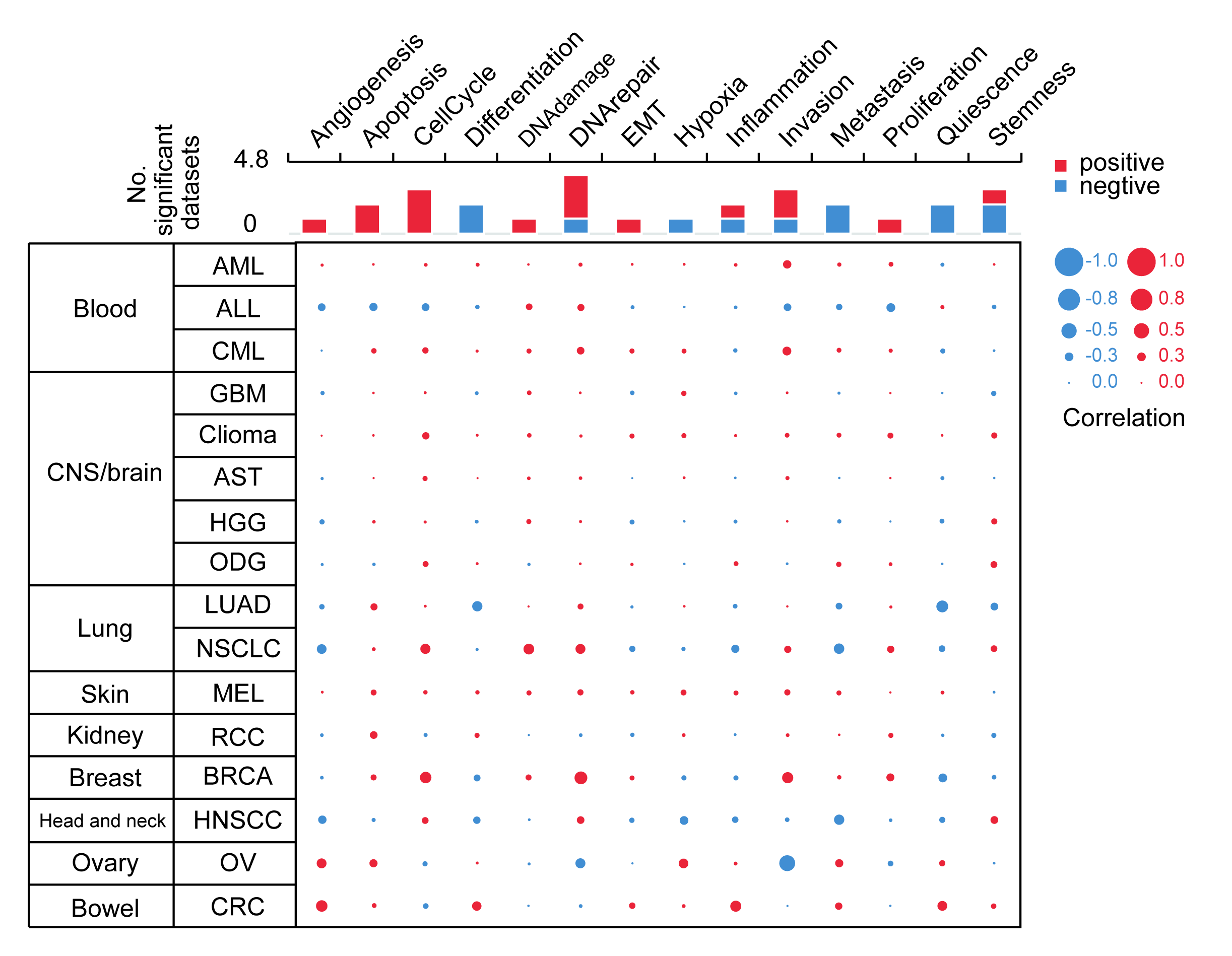

Supplement: Supplementary file 6 [file Image5.TIF]
